# High-Performance Capacitive Ultrasonic Transducer for Non-Destructive Testing of Concrete Compressive Strength

**DOI:** 10.3390/s25164903

**Published:** 2025-08-08

**Authors:** Wangyang Zhang, Jiaqian Yang, Lei Ren, Hanjie Dou, Xianglong Chen, Haoliang Jia, Yuchen Mao, Jixuan Zhang, Wanyu Xu, Hong Zhou, Xiaojing Mu

**Affiliations:** 1 Key Laboratory of Optoelectronic Technology and Systems of Ministry of Education, International Research and Development Center of Micro-Nano Systems and New Materials Technology, Chongqing University, Chongqing 400044, China; zhangwangyang@stu.cqu.edu.cn (W.Z.);; 2 Ministry of Education Key Laboratory of Micro and Nano Systems for Aerospace, School of Mechanical Engineering, Northwestern Polytechnical University, Xi’an 710072, China

**Keywords:** CMUTs, ultrasonic non-destructive testing, concrete compressive strength, time of flight

## Abstract

Ultrasonic non-destructive testing is indispensable for assessing the compressive strength of concrete and is widely utilized in concrete structure health monitoring. However, traditional ultrasonic transducers typically have a large size, narrow bandwidth, and low sensitivity, and often rely on complex circuitry, facing numerous challenges. These limitations hinder their widespread application in real-world engineering practice. To address these challenges, this study proposes the use of Capacitive Micromachined Ultrasonic Transducer (CMUT) technology for non-destructive evaluation of concrete compressive strength. CMUTs offer key advantages, including compact structure, low cost, and high sensitivity, making them well-suited for integration and real-time field applications. Through the use of COMSOL Multiphysics simulations, a strong correlation was observed between the time of flight of ultrasonic waves and concrete compressive strength. Experimental validation was conducted by performing ultrasonic measurements and standard compressive strength tests on concrete specimens. The time of the first highest-amplitude wave (T_FHAW) was extracted as a characteristic parameter and compared against the measured compressive strengths. The results demonstrate a clear linear inverse relationship between T_FHAW and compressive strength, with a coefficient of determination R^2^= 0.99, confirming the accuracy and reliability of the method. These findings suggest that CMUT-based ultrasonic testing provides an effective and precise approach for non-destructive prediction of concrete compressive strength.

## 1. Introduction

The compressive strength of concrete is a fundamental mechanical property that directly affects the safety, stability, and durability of structures [1,2]. Accurate and reliable measurement of this parameter is crucial for a wide range of engineering applications, particularly in construction, transportation infrastructure, hydraulic engineering, and nuclear power facilities [3,4]. In these industries, structural integrity is paramount, and compressive strength testing serves as a critical component of engineering quality control and risk prevention. In civil and structural engineering, insufficient compressive strength can compromise a structure’s ability to bear expected loads, leading to severe consequences such as deformation, cracking, or even catastrophic collapse [5]. This is especially critical in high-rise buildings, where inadequate strength may cause differential settlement between floors and trigger large-scale structural failure. In hydraulic engineering, concrete dams must withstand significant hydrostatic pressure [6]. Any deficiency in compressive strength can result in crack propagation and, ultimately, dam failure, posing significant threats to human life and property [7]. Similarly, in nuclear power plants and other high-risk industries, concrete infrastructure must maintain exceptional compressive strength to ensure the safety of core facilities, such as reactors, under extreme operating conditions [8,9,10]. Failure in such cases may lead to equipment damage or even nuclear leakage.

Several methods have been developed to assess concrete compressive strength. Conventional techniques include the standard specimen method, where concrete cubes or cylinders are subjected to compressive loading until failure [11,12]; static load testing, which evaluates in situ structural behavior under actual load conditions; and non-destructive testing (NDT) methods, such as the rebound hammer, sonreb, and ultrasonic pulse velocity [13,14]. Among these, ultrasonic testing stands out due to its non-destructive nature, real-time feedback, high sensitivity, adaptability, efficiency, and cost-effectiveness [15]. As such, it has emerged as a promising alternative for rapid and accurate concrete strength evaluation, especially in modern construction scenarios that demand both speed and precision [16,17].

Despite the advantages of ultrasonic testing, conventional bulk lead zirconate titanate (PZT) ultrasonic transducers are limited by their bulky size, high power consumption, and poor compatibility with integrated circuits, rendering them unsuitable for applications involving complex geometries, confined spaces, or real-time dynamic monitoring [18,19]. CMUTs have recently emerged as a viable alternative to traditional ultrasonic devices. Compared with the Piezoelectric Micromachined Ultrasonic Transducer (PMUT) and other conventional transducers, CMUTs offer several notable advantages, including enhanced electromechanical coupling coefficients, superior receiving sensitivity, and a broader bandwidth [20,21]. These characteristics have facilitated their widespread adoption in various applications, such as three-dimensional ultrasonic imaging, graphical printing, and distance measurement [22,23]. However, their potential application in the measurement of concrete compressive strength remains largely underexplored.

In this study, we propose a novel ultrasonic measurement technique for assessing concrete compressive strength utilizing CMUT technology. By exploiting the compact size of CMUTs, sensors are directly affixed to opposite sides of a concrete specimen, enabling the transmission and reception of ultrasonic signals. The time of flight (TOF) of the ultrasonic waves is employed as a key parameter to evaluate the compressive strength of the concrete. A finite element simulation is conducted to model and validate the correlation between TOF and compressive strength, providing a theoretical foundation for the proposed method. This highly integrated, real-time, and accurate measurement approach offers significant potential for in situ monitoring of concrete strength in complex and confined environments. It is anticipated to improve the efficiency, accuracy, and applicability of structural health monitoring in future infrastructure projects.

## 2. Materials and Methods

The ultrasonic pulse velocity (UPV) in concrete materials primarily depends on their elastic modulus. Since the elastic modulus is closely related to the mechanical strength of a material, it is reasonable to infer that the pulse velocity may also correlate with the material’s compressive strength [24]. However, this correlation is not unique and is significantly influenced by factors such as the mix proportions, type of cement, and type of aggregate used. Therefore, the UPV method can be employed to estimate the compressive strength, provided that a specific calibration curve is established for each evaluated material.

**Figure 1:**
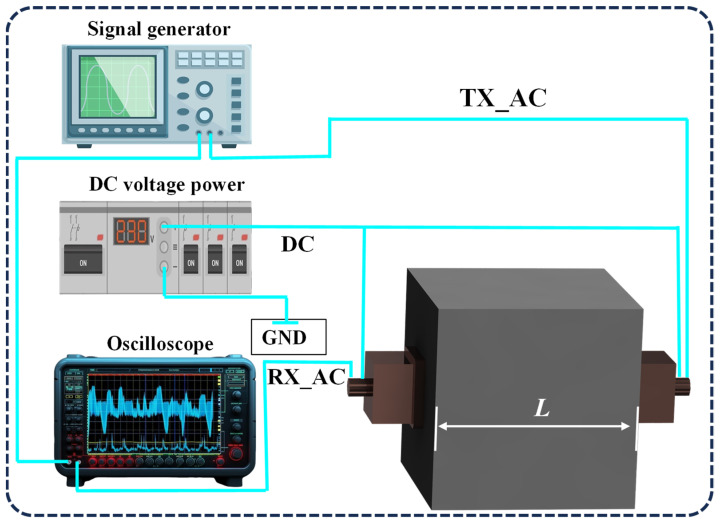
Test schematic.

The fundamental principle of the UPV test lies in measuring the propagation velocity of ultrasonic pulses through the material. As shown in Figure 1, the concrete ultrasonic flight-time detection system consists of a signal generator, a DC power supply, an oscilloscope, and an ultrasonic transducer. The signal generator (DG-1032Z, Rigol, Beijing, China) and the DC power supply (2612A, KEITHLEY, Cleveland, OH, USA) provide AC and DC excitation voltages to the CMUTs, respectively. The received signal is displayed and recorded by an oscilloscope (MSO44, Tek-tronix, Beaverton, USA). The test device usually consists of a transmitter, a receiver, and a device that displays the transmission time. The pulse velocity is defined as follows:

(1) $$v=\frac{L}{t},$$

where v is pulse velocity, *L* is path length, and t is transit time.

### 2.1. Finite Element Numerical Simulation

#### 2.1.1. Geometrical Model

To validate the feasibility of the proposed principle for estimating the compressive strength of concrete, a finite element model (FEM) was developed using the commercial software COMSOL Multiphysics 6.2 to simulate the variation in ultrasonic time of flight in response to changes in the internal structure of concrete. In the simulation process, a multiphase ultrasonic wave velocity model for concrete proposed by Lin et al. [25] was adopted. To meet the requirements of this study, the original three-dimensional model was simplified into a two-dimensional model, thereby reducing computational complexity and improving efficiency. This simplification allows for effective parameter optimization and sensitivity analysis under limited computational resources.

Based on the Monte Carlo method and the actual mix design of the concrete specimen, the area proportion of aggregates within each particle size range was calculated. Aggregates were then randomly distributed in descending order of size to ensure accurate volume fractions and gradation. The boundary of the interfacial transition zone (ITZ) was extracted using AutoCAD 2021 software, resulting in a two-dimensional random polygonal mesoscale aggregate model consistent with the macroscopic specimen.

#### 2.1.2. Ultrasonic Excitation

To broaden the frequency bandwidth for ultrasonic signal analysis and to obtain improved echo signals [26], the following expression was adopted in this simulation:

(2) $$S_{T}(t)=S_{0}(t)\sin(2\pi f_{c}t),$$

where S_T_ represents the amplitude as a function of time, t denotes the time variable, and fc is the ultrasonic frequency. S_0_ is a Hanning window function [27], which is defined as follows:

(3) $$S_{0}(t)=0.5\times [1-\cos(\frac{2\pi t}{L})]$$

where L denotes the window length, i.e., the duration of one period.

#### 2.1.3. Medium Material

**Figure 2:**
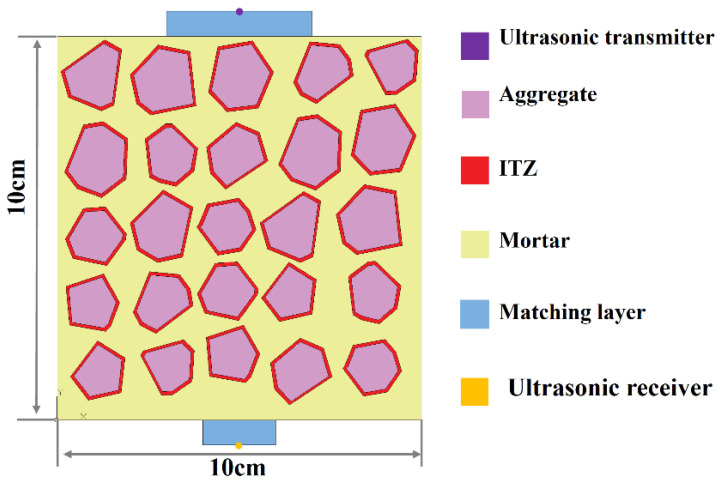
Numerical concrete model of UPV.

Considering that the propagation direction of longitudinal ultrasonic waves aligns with their particle motion direction, the internal medium of concrete in this study was classified into three distinct phases along the wave propagation path: the mortar phase, the ITZ phase, and the phase comprising both the matching layer and coarse aggregates [28]. Based on this classification, a two-dimensional simplified model was constructed, as illustrated in Figure 2.

The aggregate gradation curve plays a critical role in determining the compressive strength of concrete, particularly in the design and performance optimization of concrete mixtures. In practical engineering applications, aggregate size distribution is typically described by mass percentage [29]. Seven concretes were prepared using the mix proportions shown in Table 1, with compressive strength grades ranging from C20 to C50, increasing in increments of C5. However, in 2D numerical simulations, this representation is not directly applicable because simulations are usually based on volume fractions. Therefore, the volume fraction was calculated from the mass fraction and density, and a random aggregate distribution model was used to generate aggregates with predetermined volume fractions. In the present model, the aggregate size was specified within the range of 10 mm to 20 mm. ITZ in concrete refers to the thin layer located between the cementitious matrix and the surface of the aggregate particles [30]. This zone, typically measuring tens to hundreds of micrometers in thickness, significantly influences the overall performance of concrete. The physical and mechanical properties of the ITZ directly affect the strength, durability, and toughness of the composite material. The matching layer in the simulation is formed by applying vacuum silicone grease between the ultrasonic sensor and the surface of the concrete specimen during testing. This coupling medium serves to eliminate air gaps and reduce acoustic impedance mismatches, thereby enhancing the efficiency of ultrasonic wave transmission at the interface. Ensuring intimate contact between the transducer and the specimen via the silicone grease significantly enhances signal fidelity and measurement precision, which are critical for the accurate detection and characterization of internal structural features within concrete.

### 2.2. Simulation Validation Results

**Figure 3:**
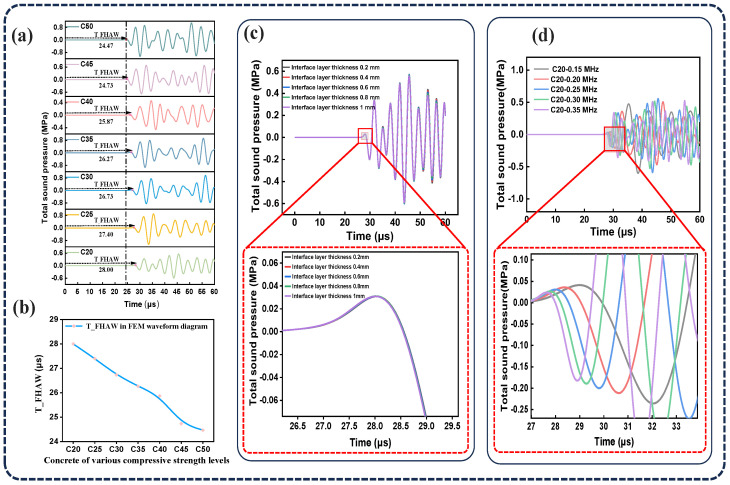
This figure presents the simulated ultrasonic TOF waveforms and key parameter variations. (**a**) TOF waveforms for concrete grades from C20 to C50. (**b**) T_FHAW in FEM simulations for different concrete grades. (**c**) TOF waveforms of C20 concrete with varying ITZ thicknesses. (**d**) TOF waveforms of C20 concrete at different emission frequencies.

**Figure 4:**
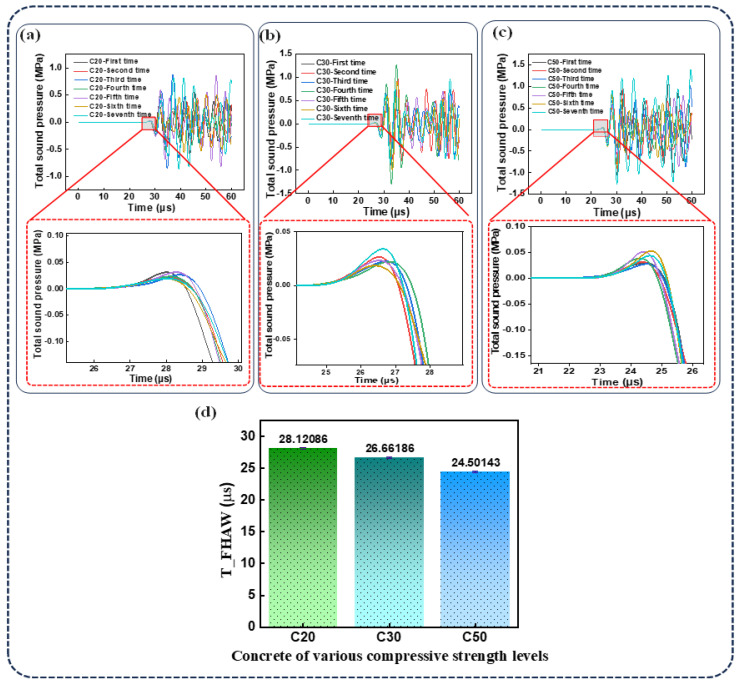
Random arrangement of aggregates in concrete. (**a**–**c**) Simulated TOF waveforms of concrete with different aggregate configurations for C20, C30, and C50 compressive strength grades. (**d**) Error bars representing the arrival time variation in the highest-amplitude wave in FEM simulations for C20, C30, and C50, indicating the influence of aggregate spatial distribution on ultrasonic wave propagation.

Based on the FEM, the relationship between TOF and concrete compressive strength was systematically investigated. Figure 3a,b illustrate the changes in arrival time within the FEM waveforms for concrete grades ranging from C20 to C50. Notably, the time of the first highest-amplitude wave (T_FHAW) significantly decreases as the concrete grade increases, indicating a strong correlation between wave propagation characteristics and material strength. High-strength concrete typically exhibits greater density and lower porosity, which result in a higher elastic modulus and reduced resistance to ultrasonic wave propagation. Consequently, the wave travels faster, leading to a shorter time of flight (TOF). In contrast, low-strength concrete often contains more internal voids and exhibits lower compaction, contributing to a lower elastic modulus, slower wave velocity, and longer TOF. Therefore, ultrasonic testing serves as an effective indirect method for estimating the compressive strength of concrete. The effects of ITZ thickness and ultrasonic frequency on the time of the first highest-amplitude wave (T_FHAW) were further examined. Initially, the ultrasonic frequency was held constant while varying the ITZ thickness within C20 concrete specimens. As illustrated in Figure 3c, when the ITZ thickness increased from 0.2 mm to 1 mm, the T_FHAW curves exhibited substantial overlap at the examined scale, suggesting that the TOF is largely insensitive to variations in ITZ thickness within this range. Subsequently, the ultrasonic emission frequency was increased from 0.15 MHz to 0.35 MHz, while keeping all other parameters constant, to analyze its effect on the time of the first peak-amplitude wave (T_FHAW) in C20 concrete. As shown in Figure 3d, the propagation time through the concrete decreases with increasing emission frequency. This phenomenon occurs because high-frequency ultrasonic waves have shorter wavelengths and faster propagation speeds, thereby reducing the time required to travel through the concrete. Additionally, high-frequency waves can penetrate the concrete more effectively and are less affected by energy loss due to pores and microcracks, thereby improving wave transmission efficiency and speed. Furthermore, the effect of varying spatial distributions of aggregates on the ultrasonic TOF was systematically investigated. For this purpose, we randomly rearranged the aggregate distributions in concrete specimens with compressive strength grades of C20, C30, and C50, each repeated seven times. As shown in Figure 4a–c, the simulation results demonstrate that the time of flight (TOF) fluctuates across seven iterations for each concrete grade, with no consistent pattern observed. Relatively larger variations are seen in lower-strength concrete, indicating greater uncertainty in wave propagation at lower compressive strength levels. The standard deviations of T_FHAW for C20, C30, and C50 are 0.15 μs, 0.14 μs, and 0.12 μs, respectively, as illustrated in Figure 4d. These findings suggest that even within the same grade of concrete, differences in internal structural arrangement can influence ultrasonic wave propagation, leading to a certain degree of variation in the estimated compressive strength.

## 3. Design and Preparation of CMUT Chip

**Figure 5:**
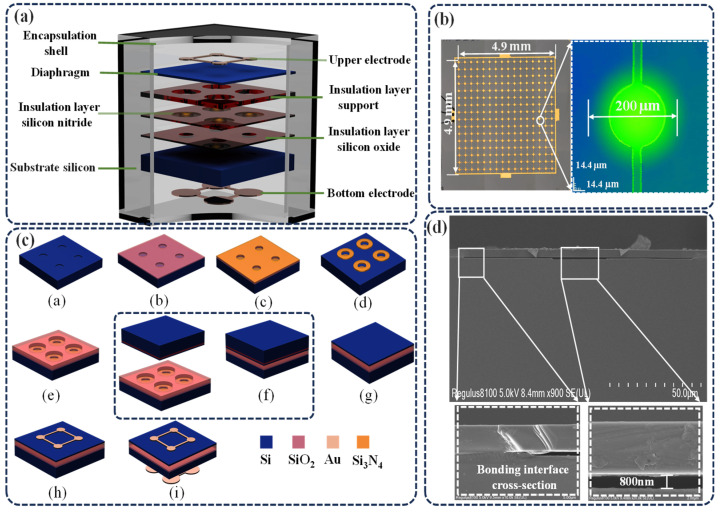
CMUT design and characterization. (**a**) Structural diagram. (**b**) Surface morphology diagram of the device. (**c**) Process flow diagram. (**d**) SEM image of the cross-section of the unit device structure.

To facilitate the measurement of concrete compressive strength, a CMUT chip was first designed and fabricated. The schematic structure of the CMUT is shown in Figure 5a. The CMUT consists of a Au top electrode, a silicon membrane, SiO_2_ and silicon nitride insulating layers, a silicon substrate, and a Au bottom electrode. Figure 5b presents the optical microscopy image of the CMUT chip, which is composed of a 16 × 16 array of elements, with a total size of 4.9mm × 4.9 mm. For application in concrete compressive strength testing, the CMUT was designed with a post-packaging resonant frequency in the range of 200–300 kHz.

Considering both device performance and fabrication feasibility, the membrane was designed with a diameter of 200 μm and a thickness of 2.5 μm, while the cavity depth was set at 0.8 μm. The maximum allowable bias voltage is 120 V. The top electrode (Ti/Au) has a diameter of 100 μm and a thickness of 150 nm, covering the vibrating region of the silicon membrane. To prevent electrical shorting between the top and bottom electrodes, insulating layers of 0.1 μm SiO_2_ and 0.15 μm Si_3_N_4_ were deposited at the cavity bottom. Additionally, to ensure high-quality Si-SiO_2_ bonding, the micropore pitch was reduced to 200 μm, thereby maximizing the fill factor and enhancing CMUT performance.

A silicon substrate with a resistivity of <15–25 Ω·cm and a thickness of 625 μm was selected. A 0.15 μm thick Au layer was deposited on the backside of the Si substrate to serve as the bottom electrode for bonding to the printed circuit board. The fabrication process steps are illustrated in Figure 5c. Lightly doped silicon wafers were used as substrates and underwent standard RCA cleaning. Photolithography was then used for patterning, followed by deep reactive ion etching (DRIE) to form uniform cavities (Step a). SiO_2_ and Si_3_N_4_ insulating layers were deposited by dry thermal oxidation and low-pressure chemical vapor deposition (LPCVD), respectively. The Si_3_N_4_ layer provides both insulation and support for secondary oxidation to form cavities (Step b). Dry etching was applied to the Si_3_N_4_ layer, and the underlying SiO_2_ was wet-etched using a buffered oxide etch (BOE) solution (Step c). A combination of dry and wet thermal oxidation was then used to form the SiO_2_ support pillars (Step d). High-temperature wafer bonding at 400 °C was performed to bond the device silicon wafer to a silicon-on-insulator (SOI) wafer (Step e). The handle layer of the SOI wafer was removed using mechanical thinning and DRIE, followed by wet etching of the SiO_2_ layer using BOE (Step f). The top electrode (Ti/Au) was deposited by physical vapor deposition and patterned by photolithography and ion beam etching (Steps g–h). Finally, the Si substrate was thinned, and a bottom Ti/Au electrode was deposited (Step i). Through these steps, a high-frequency CMUT was successfully fabricated. Subsequent experiments were performed to quantitatively assess the electromechanical coupling coefficient as well as the transmission and reception characteristics of the CMUT devices. Figure 5d presents a cross-sectional scanning electron microscope (SEM) image of the fabricated device, in which the overall cavity structure exhibits a well-defined “T” shape. The silicon-to-silicon bonding interface shows excellent quality, with no observable defects or voids, reflecting a high level of process control. The measured cavity height is 800 nm, precisely matching the design target, and other key structural parameters are also in close agreement with the specified design values.

## 4. Encapsulation of the CMUT Chip

**Figure 6:**
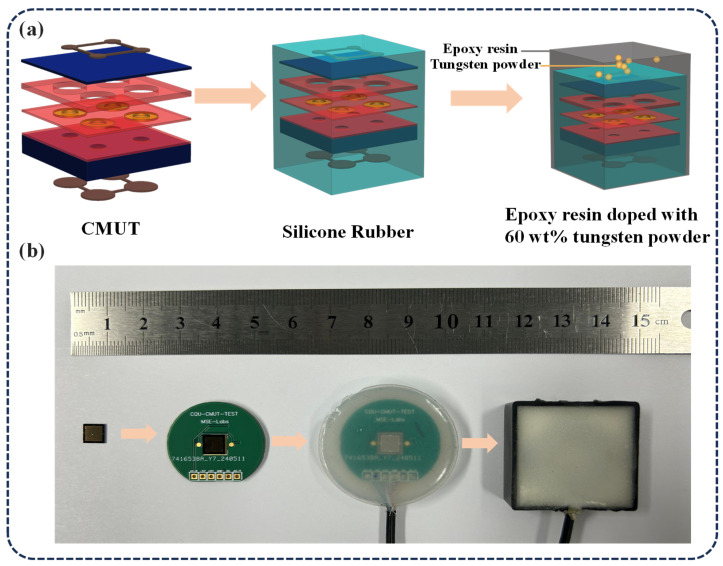
CMUT packaging. (**a**) CMUT packaging process diagram. (**b**) Real-object process flow diagram.

To protect the CMUT from external environmental factors and to prevent mechanical damage or corrosion during operation, an encapsulation technique was implemented. To further improve acoustic coupling between the CMUTs and concrete, matching layers were introduced to adjust acoustic impedance, minimize signal reflection, and enhance ultrasonic transmission. As illustrated in Figure 6, a multilayer encapsulation strategy was adopted, combining silicone rubber and tungsten-doped epoxy resin to optimize CMUT performance under complex environmental conditions. The encapsulation process consists of three key steps: (1) accurately weighing and mixing 40 wt% epoxy resin with 60 wt% tungsten powder (particle size: 10 μm) in a beaker [31]; (2) placing the mixture in a vacuum chamber at 0.08 MPa to remove air bubbles; and (3) pouring the degassed mixture into a mold and curing it at room temperature. This encapsulation approach significantly enhances the mechanical durability and environmental robustness of the CMUT, thereby improving its reliability for ultrasonic testing applications in concrete.

## 5. Performance Testing of the CMUT Chip

**Figure 7:**
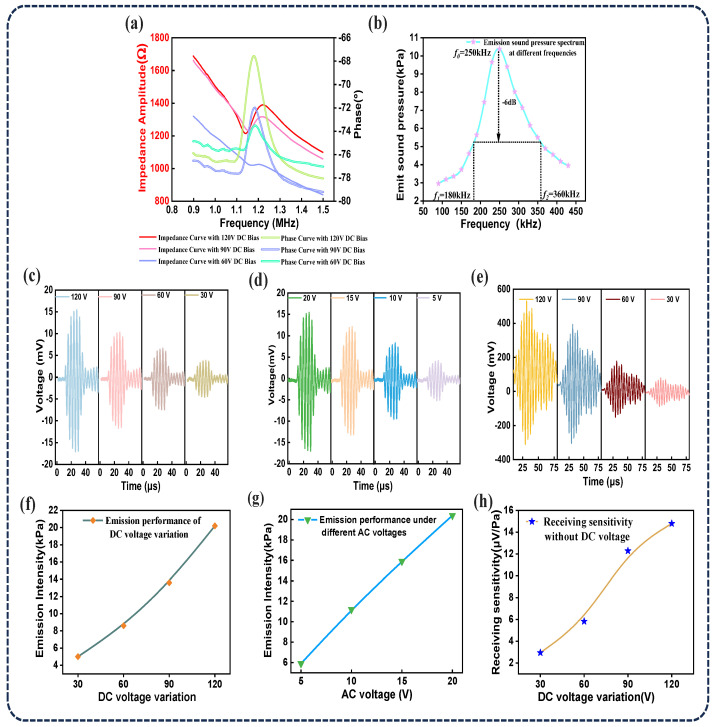
Performance testing of the CMUT chip. (**a**) CMUT impedance curve. (**b**) Emission sound pressure spectrum. (**c**,**f**) Emission performance of CMUT chip under different DC voltages. (**d**,**g**) Transmitting performance of CMUT chip under different AC voltages. (**e**,**h**) CMUT chip receiving performance under different DC voltages.

The impedance–phase curve of the unencapsulated CMUT chip was measured using an impedance analyzer (E4990A, Agilent Technologies, Santa Clara, USA). This measurement was essential for determining the resonance frequency of the CMUT in air and for calculating parameters of potential impedance matching components. As shown in Figure 7a, under a DC bias of 120 V, the resonance and anti-resonance frequencies of the CMUT were found to be 1.21 MHz and 1.38 MHz, respectively, yielding an electromechanical coupling coefficient of 23.12%.

Experimental testing was conducted using a commercial needle-type hydrophone (NH2000, Precision Acoustics, Dorchester, UK). The results demonstrated that the CMUT exhibited a −6 dB bandwidth of 70.4% at a 10 mm distance in deionized water (corresponding to the point of maximum acoustic pressure output under identical DC and AC driving conditions), as shown in Figure 7b. Compared to the unencapsulated CMUT, the multilayer-encapsulated CMUT using silicone rubber and tungsten-doped epoxy resin showed a slightly narrower bandwidth. However, in specific application scenarios, a reduced bandwidth can offer benefits such as minimized noise interference, improved anti-interference performance, and enhanced system stability. Moreover, the multilayer encapsulation contributes to better overall transducer performance and environmental adaptability.

Figure 7c,d,f,g illustrate the variation in axial transmission performance of the CMUT under different DC and AC driving voltages. The test results showed that, with the AC voltage fixed at 20 V, the transmitted acoustic pressure increased as the DC bias voltage increased, reaching a maximum of 20.2 kPa at 120 V. In contrast, when the DC bias was fixed at 120 V, the transmitted acoustic pressure decreased with increasing AC driving voltage. Figure 7e,h show that the CMUT’s receiving sensitivity increased with DC bias, reaching a peak value of 14.79 μV/Pa. For optimal voltage response in concrete TOF detection, two ultrasonic transducers were positioned on opposite sides of a 10 cm thick concrete block. One transducer was driven to transmit, while the CMUT—biased at 90 V—was used as the receiver. Experiments conducted in silicone oil revealed that the CMUT produced the highest output voltage at a resonance frequency of 0.25 MHz. This resonance frequency matched well with the designed frequency range (0.2–0.3 MHz), indicating good alignment between design parameters and actual operating conditions. Therefore, in subsequent concrete testing, the operating frequency of the CMUT was set to 0.25 MHz.

## 6. Ultrasonic Time-of-Flight Testing in Concrete

To verify the feasibility of using CMUT technology for ultrasonic non-destructive testing of concrete compressive strength, experiments were conducted on concrete specimens of different strength grades while maintaining consistent specimen dimensions (10 cm × 10 cm × 10 cm). All experiments were conducted at a constant ambient temperature of 26 °C. During the experiments, the ultrasonic transducer was driven using an AC excitation of 10 V at a frequency of 0.25 MHz, with five pulses per excitation group. The transmission interval was set to 1 ms. To enhance measurement accuracy, continuous ultrasonic excitation was employed, and the average waveform was extracted from multiple signals acquired by the oscilloscope. High-vacuum silicone grease was used to ensure firm coupling of the CMUTs to the central regions on both sides of the concrete specimens. In operation, the centers of the transmitting and receiving ultrasonic transducers were aligned as closely as possible along the same horizontal axis. The transmitting transducer emitted ultrasonic signals into the concrete specimen, which were then detected and received by the receiving transducer during propagation.

**Figure 8:**
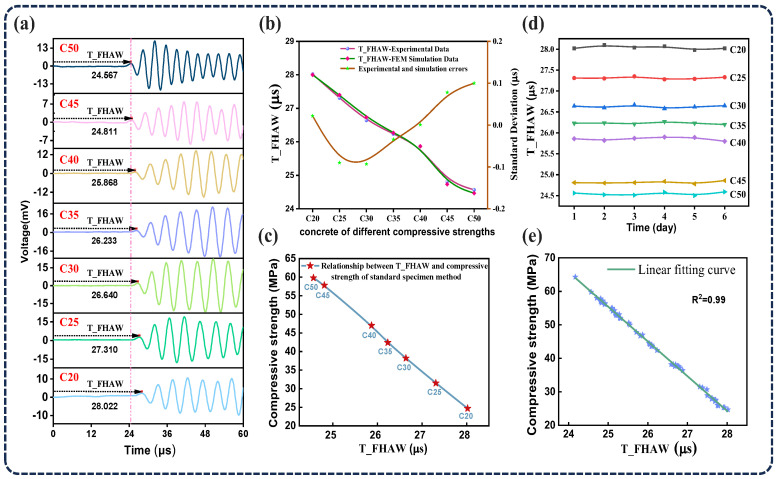
Analysis of the ultrasonic time of flight for concrete samples with different compressive strength grades. (**a**) T_FHAW waveforms for concrete grades C20 to C50. (**b**) Error analysis between simulated and experimental T_FHAW results. (**c**) Correlation between T_FHAW measurements and compressive strength obtained from standard testing methods. (**d**) Results from six repeated trials demonstrating the repeatability of the measurements. (**e**) Statistical analysis of T_FHAW values from ultrasonic measurements and compressive strength obtained from standard testing methods for multiple concrete samples.

The experimental results of ultrasonic TOF measurements are shown in Figure 8a. For concrete with a compressive strength grade of C20, the time of T_FHAW is observed to be the longest. As the compressive strength grade increases, T_FHAW progressively decreases. This trend reflects the higher density and elastic modulus associated with increased concrete strength, which directly enhance ultrasonic wave velocity and consequently reduce propagation time. These experimental observations align closely with the results obtained from finite element simulations. As described in Figure 8b, we extracted the T_FHAW from both the FEM simulations and ultrasonic testing data. The comparison shows excellent agreement, with a maximum deviation within ±0.1 μs, which confirms the reliability of the finite element modeling in replicating the actual wave propagation behavior. The same specimens were then subjected to standard destructive compressive strength testing. The compressive strength values of C20 to C50 concretes were obtained through destructive compression tests on standard cube specimens with dimensions of 10 cm × 10 cm × 10 cm. The tests were conducted in accordance with the Chinese national standard GB/T 50081-2019: Standard for Test Methods of Mechanical Properties of Ordinary Concrete [32]. A hydraulic universal testing machine was used to apply the load, and the maximum load at failure was recorded to calculate the compressive strength. As illustrated in Figure 8c, a clear linear relationship was observed between the T_FHAW values obtained using CMUT-based ultrasonic measurements and the compressive strength values determined by the standard method for C20 to C50 concretes. This validates the feasibility of estimating compressive strength using CMUTs. The repeatability of the ultrasonic flight-time measurements was also confirmed, as shown in Figure 8d. Six repeated tests were conducted for each strength grade, with minimal variation in the received flight times. The root mean square error (RMSE) of the received flight times for the C20 to C50 concrete grades were 0.04215 μs, 0.02608 μs, 0.03327 μs, 0.02449 μs, 0.03933 μs, 0.02875 μs, and 0.0383 μs, respectively—all within 3% of the average received signal, indicating excellent repeatability. In addition, a statistical analysis was conducted between the T_FHAW measurements and the compressive strength values obtained from standard destructive tests. As shown in Figure 8e, a strong correlation was observed between T_FHAW and compressive strength, with an R^2^ value of 0.99. This strong correlation indicates that the T_FHAW measured by the CMUT is a promising non-destructive parameter for estimating the compressive strength of concrete. The results of this study demonstrate that integrating CMUT-based ultrasonic measurements into concrete structural health monitoring systems is a feasible approach to enhance durability assessment and enable predictive maintenance strategies. Compared with conventional PZT-based detection methods, our CMUT demonstrates superior performance in terms of linear detection range and correlation. The results are summarized in Table 2.

## 7. Conclusions

In conclusion, we have successfully demonstrated a technique for evaluating the compressive strength of concrete using a CMUT. In our experimental setup, a pair of ultrasonic transducers was coupled to both sides of the concrete specimen using a vacuum resin coupling agent to ensure stable sound transmission. One transducer served as the transmitter, while the other acted as the receiver. The fundamental principle of this method lies in the variation in ultrasonic propagation speed through concrete with different compressive strengths, which directly affects the measured time of flight. Stronger concrete, characterized by a higher density and elastic modulus, allows faster ultrasonic wave transmission, thereby reducing the TOF. To validate this principle, a two-dimensional finite element model was established, and the simulated T_FHAW values were compared with those obtained from ultrasonic testing. The results demonstrate that, within the compressive strength range of C20 to C50, T_FHAW decreases consistently as the strength increases. This behavior exhibits strong concordance with both theoretical predictions and numerical simulation outcomes. In addition, destructive tests were conducted using the standard specimen method to obtain compressive strength data, which were then analyzed in comparison with the T_FHAW values measured by the CMUT-based ultrasonic transducer. The analysis revealed a strong linear correlation between T_FHAW and compressive strength (R^2^ = 0.99), further confirming the effectiveness of the proposed method. This study introduces a compact, non-destructive, and highly reliable method for concrete strength assessment, offering a promising alternative to traditional destructive testing methods. The CMUT-based system not only achieves high sensitivity and repeatability but also lends itself to integration into real-time structural health monitoring platforms. With further optimization, this technique has the potential to be deployed in field applications for infrastructure maintenance, safety evaluations, and durability forecasting, especially in environments that are difficult to access or require continuous monitoring. Overall, these capabilities represent a substantial advancement toward intelligent, predictive maintenance strategies within the field of civil engineering.

## Figures and Tables

**Table 1:** Concrete mix proportions for different strength grades.

Strength Grade	Water (Kg/m^3^)	Cement (Kg/m^3^)	Fly Ash (Kg/m^3^)	Fine Aggregate (Kg/m^3^)	Superplasticizer (Kg/m^3^)	Coarse Aggregate (Kg/m^3^)
C20	165	200	80	850	3.92	1090
C25	163	220	90	820	4.65	1097
C30	165	250	80	785	5.28	1120
C35	165	285	90	734	6.75	1126
C40	160	320	95	690	7.88	1147
C45	155	350	95	640	8.9	1167
C50	150	410	85	597	10.89	1188

**Table 2:** Performance comparison between CMUT and conventional PZT-based NDT systems for concrete compressive strength evaluation.

Test Method	Production Materials	Linear Range	Linear Coefficient R^2^	Ref
PZT Bulk	Lead Zirconate Titanate (toxic)	C15–C35	0.71	[33]
PZT Bulk	Lead Zirconate Titanate (toxic)	C20–C50	0.88	[34]
PZT Bulk	Lead Zirconate Titanate (toxic)	C10–C35	0.951	[35]
PZT Bulk	Lead Zirconate Titanate (toxic)	C10–C35	0.9453	[36]
PZT Bulk	Lead Zirconate Titanate (toxic)	C20–C65	0.3063	[37]
CMUT	Si (non-toxic)	C20–C50	0.99	This work

## Data Availability

The data presented in this study are available on request from the corresponding author.
